# In Situ Growth of CdZnS Nanoparticles@Ti_3_C_2_T*_x_* MXene Nanosheet Heterojunctions for Boosted Visible-Light-Driven Photocatalytic Hydrogen Evolution

**DOI:** 10.3390/nano13152261

**Published:** 2023-08-06

**Authors:** Zelin Li, Yang Zhao, Qinglin Deng, Xuhui Zhu, Yipeng Tan, Ziwen Feng, Hao Ji, Shan Zhang, Lingmin Yao

**Affiliations:** 1 School of Physics and Materials Science, Guangzhou University, Guangzhou 510006, China; zl-li@foxmail.com (Z.L.); zy843795537@163.com (Y.Z.); zxh2674091377@foxmail.com (X.Z.); a1693433393@outlook.com (Y.T.); ziwenfong@foxmail.com (Z.F.); jihao0114@foxmail.com (H.J.); lingminyao@gzhu.edu.cn (L.Y.); 2 Research Center for Advanced Information Materials (CAIM), Huangpu Research & Graduate School of Guangzhou University, Guangzhou 510555, China

**Keywords:** CdZnS, Mxene, photocatalyst, heterojunction, hydrogen evolution

## Abstract

Using natural light energy to convert water into hydrogen is of great significance to solving energy shortages and environmental pollution. Due to the rapid recombination of photogenerated carriers after separation, the efficiency of photocatalytic hydrogen production using photocatalysts is usually very low. Here, efficient CdZnS nanoparticles@Ti_3_C_2_T*_x_* MXene nanosheet heterojunction photocatalysts have been successfully prepared by a facile in situ growth strategy. Since the CdZnS nanoparticles uniformly covered the Ti_3_C_2_T*_x_* Mxene nanosheets, the agglomeration phenomenon of CdZnS nanoparticles could be effectively inhibited, accompanied by increased Schottky barrier sites and an enhanced migration rate of photogenerated carriers. The utilization efficiency of light energy can be improved by inhibiting the recombination of photogenerated electron-hole pairs. As a result, under the visible-light-driven photocatalytic experiments, this composite achieved a high hydrogen evolution rate of 47.1 mmol h^−1^ g^−1^, which is much higher than pristine CdZnS and Mxene. The boosted photocatalytic performances can be attributed to the formed heterojunction of CdZnS nanoparticles and Ti_3_C_2_T*_x_* MXene nanosheets, as well as the weakened agglomeration effects.

## 1. Introduction

Energy shortages and environmental pollution are the main problems facing the world today [1,2]. Hydrogen, a clean and carbon-free natural energy source with a high energy density, can aid in the better resolution of energy and environmental issues [3,4,5]. Currently, hydrogen is used in many fields, such as the oil industry, the new energy industry, and so on. As the demand for hydrogen continues to increase, the scientific community is also exploring ways to increase hydrogen production [6,7,8]. Since Fujishima and Honda discovered the phenomenon of photocatalytic water splitting in 1972 [9], scientists have been working on this technology. Photocatalytic hydrogen production, as a green and pollution-free technology that can convert solar energy into hydrogen energy, has now become one of the most promising research directions.

In the past decade, metal sulfides have been widely employed in various fields, such as optical imaging and energy storage applications [10,11,12], due to their excellent electronic, optical, and semiconductor properties. Transition metal sulfides, such as WS_2_, MoS_2_, ZnS, and CdS, are widely cited in the field of photocatalysis due to their suitable band gaps [13]. Ganapathy et al. [14] prepared amorphous SrTiO_3_-crystalline PbS heterojunctions that exhibited a hydrogen production performance of 5.9 mmol h^−1^ g^−1^ under ultraviolet light irradiation. Zhuge et al. [15] deposited molybdenum disulfide onto cadmium sulfide quantum dots, promoting charge separation and achieving a photocatalyst with hydrogen production performance as high as 13.13 mmol h^−1^ g^−1^. Among them, CdS exhibits prominent performance [16]. However, CdS suffers from poor stability and is associated with certain toxicities [17]. To overcome these issues, cadmium sulfide is typically coupled with other materials to form heterojunctions, aiming to achieve enhanced photocatalytic activity and improved photostability [18]. Zinc sulfide has a wide band gap and a similar structure to cadmium sulfide. Zinc sulfide and cadmium sulfide can form an infinitely miscible solid solution of Cd_1−*x*_Zn*_x_*S [19,20,21,22]. The band gap of Cd_1−*x*_Zn*_x_*S can be adjusted by controlling the ratio of zinc to cadmium in the range of 2.4–3.6 eV to achieve better photocatalytic stability and hydrogen production performance [23,24,25]. According to existing reports, when the ratio of zinc to cadmium is 1:1 (CdZnS, abbreviated as CZS), it has the best photocatalytic activity [23,26,27]. Moreover, the photocorrosion caused by the accumulation of photogenerated pores on the surface of single-transition metal sulfide and the recombination of photogenerated electron holes are the two main problems that affect the photocatalytic performance of CZS. At present, there are several mainstream improvement strategies, for example, loading a co-catalyst, constructing a heterojunction, coupling with nano-carbon, constructing a Z-scheme system, and so on [28]. One of the most effective strategies is to use precious metals (such as Pt, Au, etc.) as supported catalysts to facilitate the separation of photogenerated electron holes. The addition of metals with higher work functions and excellent conductivity can induce surface plasmon resonance (SPR) effects and Schottky barriers. This improves the dynamics of charge carriers through spatial confinement of electrons and accelerated migration for redox reactions [29]. However, due to the high price and low reserves, this strategy cannot be widely used; thus, a new co-catalyst is urgently needed [30,31,32].

Mxene is a two-dimensional material with the general form M*_n_*_+1_X*_n_*T*_x_*, where M, X, and T represent the transition metal, C or N, and the surface ends involve -OH, -O and -F, respectively [28,33]. Among them, Ti_3_C_2_T*_x_* is one of the most promising materials, which has abundant hydrophilic functional groups, good conductivity, and optical response ability, as well as the advantages of enhanced carrier separation [34,35,36]. Moreover, due to its metallic nature, it can potentially form a Schottky barrier with semiconductor photocatalysts, thereby capturing electrons and playing a significant role in suppressing the recombination of photogenerated electrons. Ti_3_C_2_T*_x_* can increase the surface-active sites, which is more conducive to improving the hydrogen production performance of CZS [37,38,39]. Additionally, the layered structure of Ti_3_C_2_T*_x_* can be tailored into 2D or 3D morphologies, as needed, maximizing the utilization of light energy [29]. Based on the above characteristics, scholars have carried out a lot of research on Ti_3_C_2_T*_x_* as a co-catalyst. Yin et al. [27] in situ synthesized titanium dioxide nanoparticles on a Ti_3_C_2_ matrix and then hydrothermally formed Z-scheme TiO_2_@Ti_3_C_2_/Cd_0.5_Zn_0.5_S (TO/CZS) multi-structure nanocomposites. The obtained photocatalyst has a higher degradation rate. Chen et al. [40] prepared a two-dimensional cadmium sulfide nanosheet composite Ti_3_C_2_T*_x_* photocatalyst with a higher photocatalytic activity. Tao et al. [28] reported the highest hydrogen evolution performance of CZS@Ti_3_C_2_T*_x_* materials synthesized via a hydrothermal method, reaching 29.3 mmol h^−1^ g^−1^. Zhong et al. [35] synthesized CZS/Ti_3_C_2-*A*40_ with a maximum hydrogen evolution performance of 13.44 mmol h^−1^ g^−1^. A large number of studies have shown that Ti_3_C_2_T*_x_* as a co-catalyst can improve the efficiency of carrier migration and accelerate the transfer of charge. Moreover, the combination of zinc cadmium sulfide and Ti_3_C_2_T*_x_* not only improves the photocatalytic activity of CZS, but also alleviates the photocorrosion of CZS to some extent [41].

Due to the internal electric field generated by the heterojunction generated at the interface between the metal and the semiconductor, it can play a crucial role in promoting the separation of photocurrent-carrying electrons and improving the migration efficiency [42,43,44]. Inspired by this, we successfully constructed the heterojunction composite CZS/Ti_3_C_2_T*_x_* by a hydrothermal in-situ growth and assembly method in this work. The negative charge of Ti_3_C_2_T*_x_* causes electrostatic adsorption, so cadmium and zinc ions could be tightly attached to Ti_3_C_2_T*_x_*, which effectively inhibited the agglomeration of CZS nanoparticles. In this way, the efficient separation and utilization of electrons and holes can be realized. As a result, a kind of photocatalyst with more efficient hydrogen production performance can be obtained.

## 2. Materials and Methods

### 2.1. Materials

Chemicals (all analytical grade) were purchased from Aladdin Biochemical Technology Co., Ltd. (Shanghai, China) and used without further purification. These included zinc acetate dehydrate (Zn(OCOCH_3_)_2_•2H_2_O), cadmium acetate dehydrate (Cd(OCOCH_3_)_2_•2H_2_O), thioacetamide (TAA), sodium hydroxide (NaOH), sodium sulfide (Na_2_S), and sodium sulfite (Na_2_SO_3_). Ti_3_C_2_T*_x_* was purchased from Xianfeng Nanomaterials Technology Co., Ltd. (Nanjing, China).

### 2.2. Synthesis of Photocatalysts

**Figure 1:**
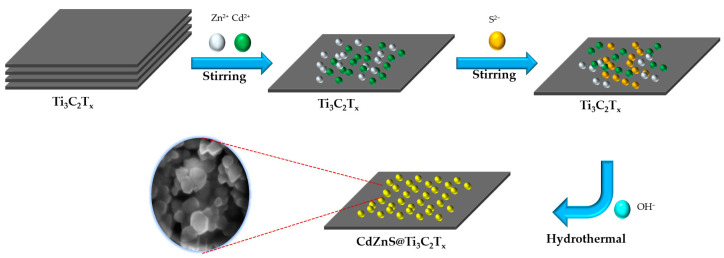
Schematic of synthesis of CdZnS/Ti_3_C_2_T*_x_*.

The CdZnS/Ti_3_C_2_T*_x_* photocatalyst was synthesized by the method shown in Figure 1. Solution A: 2 mmol of cadmium acetate hydrate and 2 mmol of zinc acetate hydrate were dissolved in 4 mL of deionized water, and then an appropriate amount of Ti_3_C_2_T*_x_* was added to the above solution. Finally, the mixture was stirred for 1 h. Solution B: 5 mmol of thioacetamide (TAA) was added to 4 mL of deionized water, then dissolved by stirring. Solution C: 8 mmol NaOH was dissolved in 2 mL of deionized water. In a typical synthesis, Solution B was added dropwise to solution A and stirred for about 1 h. Then, 2 mL of 4 mol L^−1^ NaOH aqueous solution was added, drop by drop, in the stirred state after stirring for another hour. The mixture was hydrothermally treated at 180 °C for 24 h. In this work, CdZnS samples with different ratios of Ti_3_C_2_T*_x_* were named CZS-1, CZS-2, CZS-2.5, CZS-3, and CZS-5, where the numbers represent the mass percentage of added Ti_3_C_2_T*_x_* (1%, 2%, 2.5%, 3%, and 5%). The preparation process of the original CZS is similar to that of the CZS@Ti_3_C_2_T*_x_* composite photocatalyst, with the difference being the absence of Ti_3_C_2_T*_x_* in the precursor solution of the original CZS.

### 2.3. Characterization

The structure of the prepared photocatalyst was characterized by X-ray diffraction (XRD, PW3040/60, PANalytical, Almelo, The Netherlands) with a scanning range of 10–80°. The micromorphology, lattice stripe, and bonding information of the fabricated photocatalyst heterojunction interface were analyzed using field emission scanning electron microscopy (SEM, JSM-7001F, JEOL, Akishima, Japan) and field emission transmission electron microscopy (TEM, Talos-F200S, FEI, MA, USA). The valence bands and elemental compositions of the photocatalysts were investigated using X-ray photoelectron spectroscopy (XPS, K-Alpha Nexsa, Thermal Science, USA). The light absorption performance of the photocatalyst was analyzed with a UV-VIS spectrophotometer (UV-VIS, UV-3600Plus, Shimadzu, Japan) in the range of 200 to 800 nm. The photoluminescence intensity of the samples at an excitation wavelength of 375 nm was determined using a fluorescence spectrometer (PL, FS5, Edinburgh Instruments, Livingston, UK).

### 2.4. Photoelectrochemical Measurement

Electrochemical impedance spectroscopy (EIS) and photocurrent response tests were performed using a standard three-electrode system at room temperature. A homogeneous suspension was formed by adding 10 mg of the sample to a mixture of 200 μL ethanol and 20 μL Nafion solution, followed by sonication for 30 min. The suspension was lowered onto the surface of cleaned tin fluoride oxide (FTO)-doped glass. Subsequently, the substrate was dried at 60 °C for 12 h. For recording photoresponse signals, a 300 W Xe lamp (with a UV cutoff filter, λ > 420 nm) was used as the light source. The frequency range of the electrochemical impedance spectroscopy (EIS) measurement was 10^5^ Hz to 0.1 Hz.

### 2.5. Photocatalytic H_2_ Evolution Performance

The photocatalytic hydrogen evolution reaction was conducted on an all-glass automated on-line trace gas analysis system (Labsolar-6A, PerfectLight, Beijing, China). A 10 mg quantity of photocatalyst was evenly dispersed in 100 mL of 0.35 M Na_2_S/0.25 M sodium sulfite aqueous solution and stirred using sonication for 30 min. The 300 W Xe lamp is equipped with a 420 nm cutoff filter (λ > 420 nm). Before illumination, the system was evacuated to eliminate the influence of impurities, which were detected using a gas chromatograph (GC9790II, FuLi, Zhejiang, China. TCD detector, and Ar as the carrier gas). Hydrogen production per hour was recorded. The apparent quantum yield (AQY) was measured under similar photocatalytic conditions. The difference is that AQY was measured using a 420 nm bandpass filter and the optical power density was measured using an irradiometer. AQY was calculated according to Equation (1) [45]:

(1) $$\mathrm{AQY}(\%)=\frac{\left(number\;of\;evolved\;H_{2}\;molecules*2\right)}{\left(number\;of\;incident\;photos\right)}\times 100\%$$

## 3. Results and Discussion

**Figure 2:**
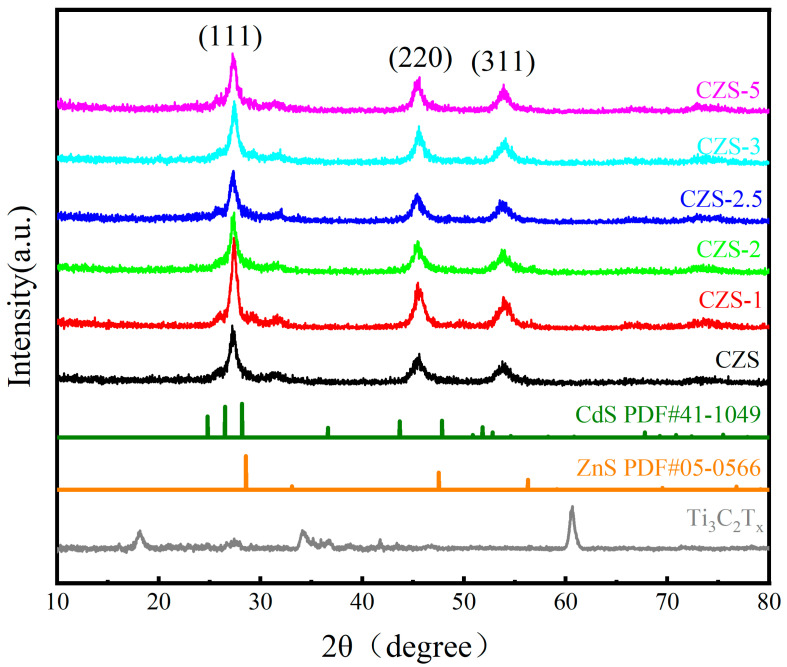
XRD patterns of as-prepared samples.

The XRD patterns of the as-prepared samples are shown in Figure 2. As can be seen, the CZS with an amphibolite structure had a distinct characteristic peak. According to the standard card, it can be indexed to the standard hexagonal phase cadmium sulfide (JCPDS 41-1049) and cubic phase zinc sulfide (JCPDS 05-0566). In contrast to them, half of the Cd atoms were replaced by Zn atoms, indicating the formation of a homogeneous solid-phase solution. It was not a mixture of cadmium sulfide and zinc sulfide on the physical layer. The peaks of the CZS/Ti_3_C_2_T*_x_* composite photocatalyst at 26.5°, 44.8°, and 52.3° can be ascribed to the (111), (220), and (311) planes of CZS, respectively, which is consistent with the XRD peak positions of CZS in previous literature [28]. The diffraction peaks of Ti_3_C_2_T*_x_* were consistent with previous literature reports [46]. Compared to cubic zinc sulfide (JCPDS 05-0566), CZS exhibited the polyphase characteristics of the hexagonal phase, with a slight shift towards lower angles. This indicates that increasing the zinc content in the CZS solid solution transformed the crystal phase from hexagonal cadmium sulfide to cubic zinc sulfide, suggesting that the formed CZS was a solid solution rather than a physical mixture of cadmium sulfide and zinc sulfide. Compared with the original CZS, no significant shift was observed on the CZS/Ti_3_C_2_T*_x_* composite photocatalyst, which indicates that the recombination of CZS and Ti_3_C_2_T*_x_* did not change the crystal orientation of CZS. The absence of Ti_3_C_2_T*_x_* in the diffraction pattern was mainly due to its low quantity and CZS covering the surface of Ti_3_C_2_T*_x_*.

**Figure 3:**
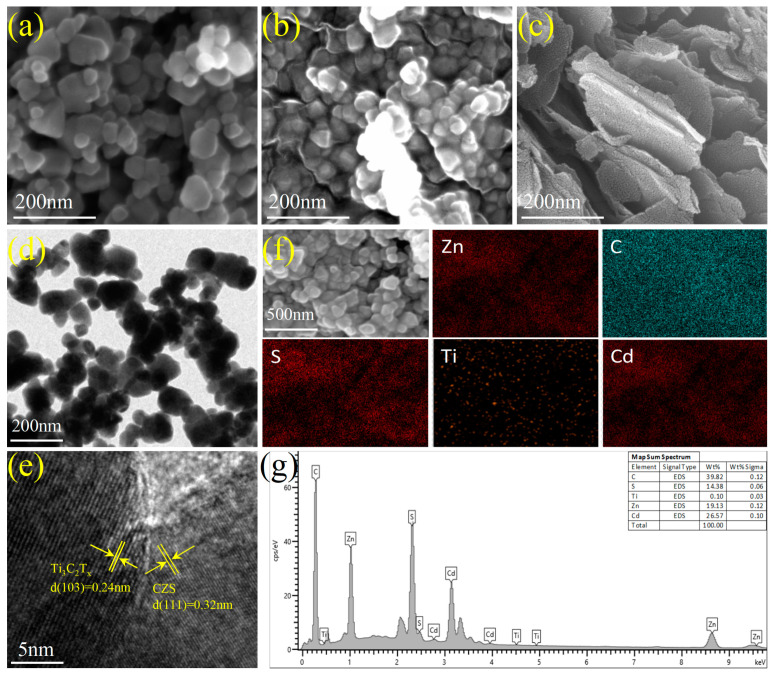
SEM images of (**a**) CZS, (**b**) CZS-2, and (**c**) Ti_3_C_2_T*_x_*; TEM images of CZS-2 (**d**,**e**); EDS mappings of CZS-2 (**f**); EDX spectra of CZS-2 (**g**).

Scanning electron microscopy (SEM) and transmission electron microscopy (TEM) were used to analyze the morphology and microstructure of the samples. The SEM and TEM images of CZS and CZS-2 are shown in Figure 3. The samples were initially synthesized as agglomerated nanoparticles (Figure 3a,d). Ti_3_C_2_T*_x_* has a unique layered structure (Figure 3c). As shown in Figure 3b, it can be seen that the CZS-2 sample had a relatively obvious structure of particles attached to the flake. CZS nanoparticles were covered on the flake Ti_3_C_2_T*_x_*. Thus, the structure had a larger contact area and stronger photoadsorption capacity. Moreover, more light reflection and light scattering on the uneven surface of the hybrid layers also improved the utilization rate of light. In addition, the gap between the layers also facilitated the entry of the sacrifice agent. Figure 3e shows two different lattices, typically 0.24 nm corresponding to the (103) plane of Ti_3_C_2_T*_x_*, and 0.32 nm corresponding to the (111) plane of CZS. The coexistence of these two lattices indicates the successful synthesis of the CZS/Ti_3_C_2_T*_x_* heterojunction photocatalyst. The uniform distribution of C, S, Zn, Cd, and Ti elements on the energy dispersive X-ray spectroscopy (EDX) mappings indicates the uniform coverage of CZS nanoparticles on Ti_3_C_2_T*_x_* (shown in Figure 3f). The similarity of the molar ratios of each element (Figure 3g) to the experimental sample further verifies the successful preparation of the sample.

**Figure 4:**
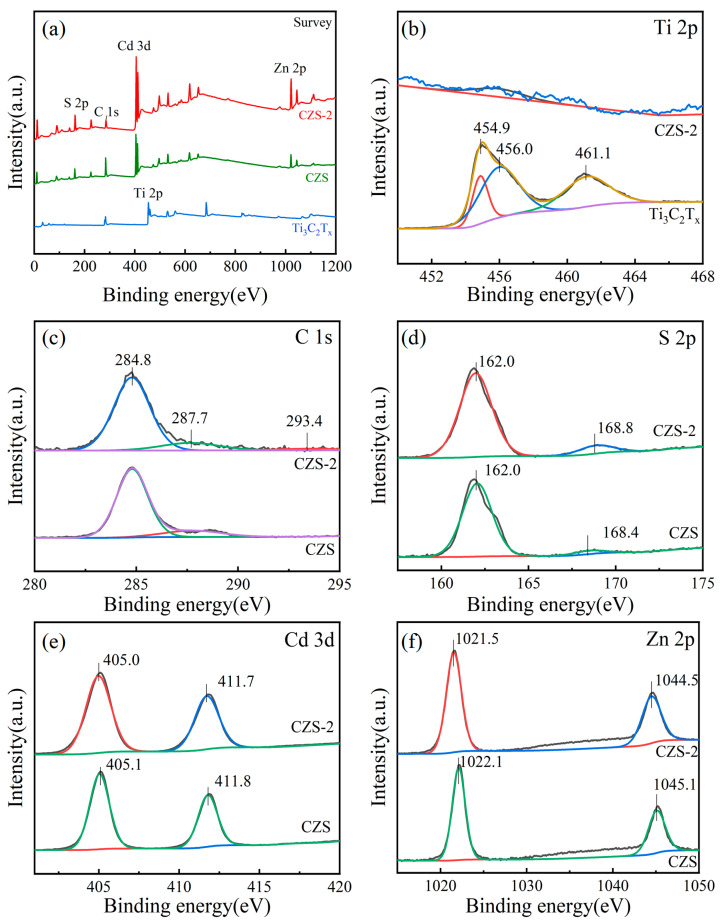
XPS spectrum of CZS, CZS-2, and Ti_3_C_2_T*_x_*; (**a**) survey, (**b**) Ti 2p, (**c**) C 1s, (**d**) S 2p, (**e**) Cd 3d, (**f**) Zn 2p.

The surface composition and chemical valence of CZS-2 were analyzed by X-ray photoelectron spectroscopy (XPS). As shown in Figure 4a, S, Zn, Cd, C, and other elements could be detected on CZS-2. In comparison with the original CZS, slight shifts in the peaks of CZS-2 could be observed, indicating a strong interaction between Ti_3_C_2_T*_x_* and CZS. Due to its extremely low content (Figure 4b) of Ti_3_C_2_T*_x_* and coverage by CZS, no obvious peak could be detected on the full spectrum of the Ti element. The peaks at 454.9, 456.0, and 461.1 eV on the pristine Ti_3_C_2_T*_x_* corresponded to Ti-C, Ti-X, and Ti-O, respectively (Figure 4b). The C 1s of CZS-2 corresponded to the C-C, C-C-O, and C-F bonds at 284.8, 287.7, and 293.4 eV, respectively (Figure 4c). Two peaks of S 2p, at 162.0 and 168.8 eV, corresponded to S 2p_3/2_ and S 2p_1/2_ (Figure 4d), respectively. Two peaks of Cd 3d at 405.0 and 411.7 eV corresponded to Cd 3d_5/2_ and Cd 3d_3/2_ (Figure 4e), respectively. Two peaks of Zn 2p at 1044.5 and 1021.5 eV corresponded to Zn 2p_3/2_ and Zn 2p_1/2_ (Figure 4f), respectively. Compared with the standard electron binding energy control, there was a slight shift in the peak positions of these elements, mainly due to the strong interaction between Ti_3_C_2_T*_x_* and CZS. The above results indicate the successful preparation of the CZS/Ti_3_C_2_T*_x_* composite photocatalyst [47].

**Figure 5:**
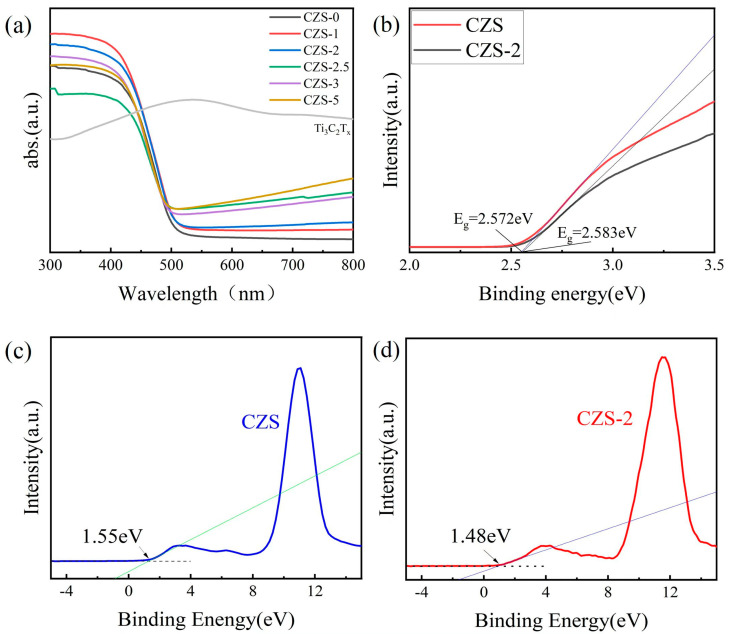
(**a**) UV-VIS DRS spectra of as-prepared samples; (**b**) Tauc plot of CZS and CZS-2. Valence band XPS spectra of (**c**) CZS and (**d**) CZS-2.

UV-VIS diffuse reflectance spectroscopy (DRS) is widely used to investigate optical absorption of photocatalysts. In order to explore effective activity of the CZS/Ti_3_C_2_T*_x_* composite photocatalyst, DRS tests were performed in the range of 300–800 nm. As shown in Figure 5a, the CZS/Ti_3_C_2_T*_x_* photocatalyst had good light absorption ability from 300 to 500 nm, and the absorption edge appeared at about 480 nm. With increasing the amount of Ti_3_C_2_T*_x_*, the light absorption gradually increased after 480 nm. In order to further study the band structure, the Kubelka–Munk equation [48] was used to calculate the band gap:

αhν = A(hν − E_g_)^1/2^ (2)

Here α, hν, and A denote the absorption coefficient, photon energy, and a constant, respectively. The calculated band gap values of CZS and CZS-2 in the original sample were 2.572 eV and 2.583 eV (Figure 5b), respectively, which also verified that CZS-2 had stronger light absorption ability. The valence band (VB) spectrum of XPS (shown in Figure 5c,d) was used to further analyze the band structure. The results show that the band gap and valence band of CZS were 2.572 eV and 1.55 eV, respectively, and the band gap and valence band of CZS-2 were 2.583 eV and 1.48 eV, respectively. The conduction band (CB) of CZS was −1.022 eV; the CB of CZS-2 was −1.103 eV according to the formula E_VB_ = E_CB_ + E_g_. Compared to CZS, CZS-2 had a more negative CB potential, which enhanced its photocatalytic reduction ability and was more conducive to the photocatalytic decomposition of the hydrogen water solution.

**Figure 6:**
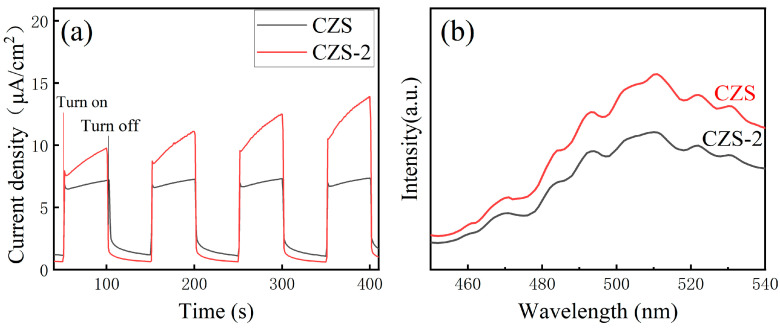
Excited state electron radioactive decay spectra (**a**), and transient photocurrent response of CZS and CZS-2 samples (**b**).

In order to further investigate the effect of Ti_3_C_2_T*_x_* on the charge transfer efficiency of CZS, the photocurrent response and photoluminescence maps were studied. As can be seen from Figure 6a, the photocurrent density of the CZS-2 composite photocatalyst was significantly higher than that of the original CZS sample, indicating that CZS had an improved photocurrent response and higher electron transfer efficiency after being combined with Ti_3_C_2_T*_x_* [49,50,51]. In Figure 6b, the PL emission intensity of the CZS-2 composite photocatalyst was also significantly lower than that of the original CZS. The lower emission intensity reflects the lower binding rate of the photoelectron-hole pair, which means that the combination of the photoelectron-hole pair could be inhibited after recombination, and the photogenerated carrier separation efficiency was higher [52,53]. This phenomenon is consistent with the photocurrent response diagram.

**Figure 7:**
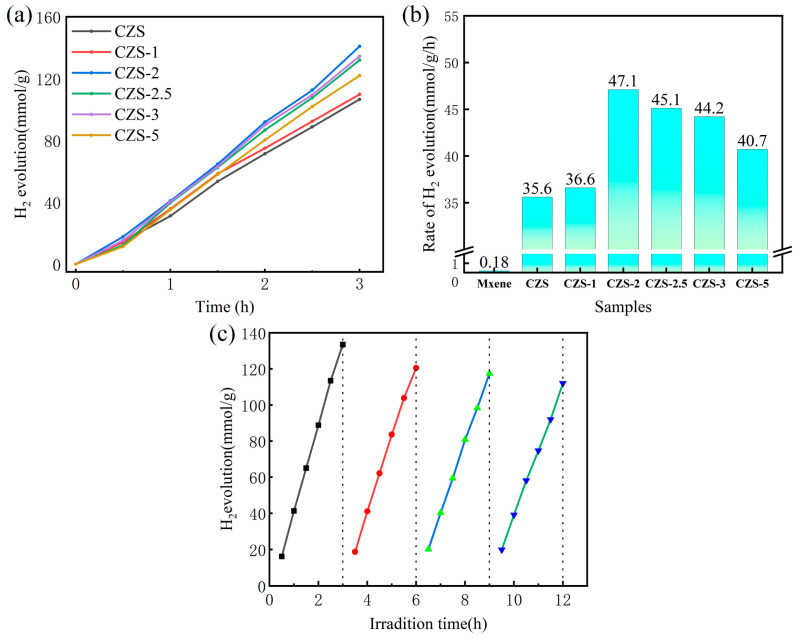
(**a**) The stability of hydrogen production performance of CZS with different Mxene mass ratios; (**b**) the rate of H_2_ evolution of CZS and CZS-*x* (*x* = 1, 2, 2.5, 3, 5) under visible light irradiation; (**c**) photocatalytic hydrogen production stability test of CZS-2 during 12 h.

Under conditions of visible illumination (UV cut-off filter, λ > 420 nm), the hydrogen evolution performance of the CZS/Ti_3_C_2_T*_x_* composite photocatalyst was analyzed. As shown in Figure 7a, CZS with different Mxene mass ratios had good stability in the hydrogen production process for 3 h. The hydrogen production performances of CZS/Ti_3_C_2_T*_x_* showed an obvious upward trend with increasing the amount of Ti_3_C_2_T*_x_* in the solution using sodium sulfide and sodium sulfite as sacrificial agents. When the content of Ti_3_C_2_T*_x_* reached 2%, the hydrogen production performance reached a maximum value of 47.1 mmol g^−1^ h^−1^, which was 1.3 times that of the original CZS sample (Figure 7b). At 420 nm, it had a higher AQY value of 27.24%. Subsequently, with increasing the amount of Ti_3_C_2_T*_x_*, the hydrogen production performance gradually decreased, indicating that excessive addition of Ti_3_C_2_T*_x_* may have a negative impact on the photocatalytic activity of CZS. An excessive amount of Ti_3_C_2_T*_x_* can result in a decrease in the concentration of the main photocatalyst CZS and also hinder the penetration of light, thereby impeding the utilization efficiency of CZS for light. Therefore, the optimum loading for Ti_3_C_2_T*_x_* is 2 wt.%. The hydrogen evolution stability of CZS/Ti_3_C_2_T*_x_* was analyzed by four repeated experiments. After four cycles of 3 h (as shown in Figure 7c), the hydrogen evolution performance of CZS/Ti_3_C_2_T*_x_* decreased slightly and the hydrogen production efficiency was 82.7%, as compared with that of the first cycle due to part of the photocatalyst covering the surface of the reaction vessel with hydrogen production, thus affecting the incident photon quantity.

**Figure 8:**
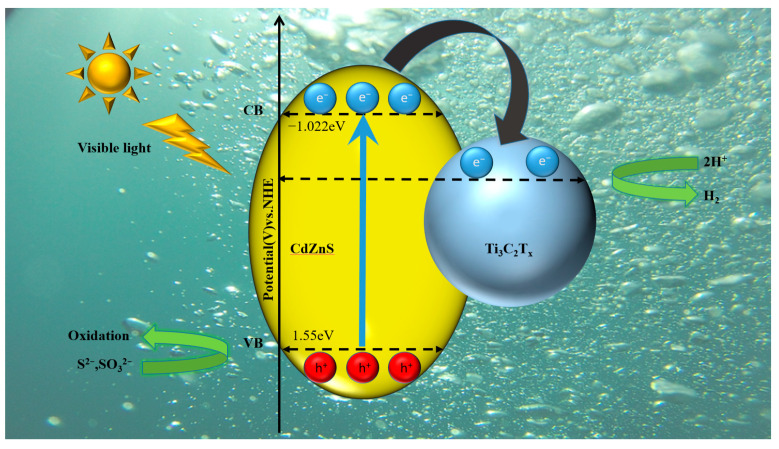
The possible photocatalytic hydrogen evolution mechanism in CdZnS/Ti_3_C_2_T*_x_* system.

Figure 8 shows the possible photocatalytic hydrogen evolution mechanism in the CZS/Ti_3_C_2_T*_x_* system. For the primitive CZS, when exposed to visible light (λ > 420 nm) after excitation, the photogenerated electrons on VB were induced to transfer to CB, then combine with H^+^ in water to produce hydrogen. The holes generated by the transfer of electrons on VB reacted with the sacrificial agents S^2−^ and SO_3_^2−^ on the surface of the photocatalyst. In the absence of Ti_3_C_2_T*_x_* recombination, the photolithogenic holes and electrons showed a high and rapid recombination rate. After adding the appropriate quantity of Ti_3_C_2_T*_x_*, a heterojunction could be formed. The Schottky barrier was generated on the CZS/Ti_3_C_2_T*_x_* interface. Additionally, due to the higher Fermi energy level of Ti_3_C_2_T*_x_* compared to CZS, photogenerated electrons tended to migrate from the conduction band of CZS to Ti_3_C_2_T*_x_*, effectively impeding the recombination of photogenerated electron-hole pairs and thereby enhancing the separation efficiency of electron-hole pairs. In addition, owing to the large dispersion area of Mxene, the agglomeration of CdZnS nanoparticles could be effectively inhibited. As a result, with the aid of Ti_3_C_2_T*_x_* Mxene, CZS showed an improved visible-light-driven photocatalytic hydrogen evolution.

## 4. Conclusions

In summary, we have constructed and characterized an efficient photocatalyst of CZS nanoparticles overlaid on cocatalyst Ti_3_C_2_T*_x_* nanosheets using an in situ growth method. The photocatalytic hydrogen production performance of CZS can be effectively improved by adding an appropriate amount of Ti_3_C_2_T*_x_* (2% mass ratio), which can construct heterojunctions to block the recombination of electrons and holes induced by photogeneration. Under visible light irradiation, it reached a high photocatalytic hydrogen production value of 47.1 mmol g^−1^ h^−1^. It could also maintain a relatively stable hydrogen production over a long period of catalysis. The main reason for the success of the modification is that the Schottky barrier is conducive to the diffusion of electrons and inhibits the recombination of electron-hole pairs induced by photogeneration. At the same time, the zinc cadmium sulfide nanoparticles uniformly covered the Ti_3_C_2_T*_x_* nanosheets, also improving the utilization rate of light and relieving the agglomeration effect of the particles, thereby achieving a high hydrogen production performance. This work can provide some valuable guidance and ideas for selecting cocatalyst modification of CZS to improve the separation of electron-hole pairs and inhibit the recombination of electron-hole pairs.

## Data Availability

Not applicable.
